# Triptolide suppresses the *in vitro* and *in vivo* growth of lung cancer cells by targeting hyaluronan-CD44/RHAMM signaling

**DOI:** 10.18632/oncotarget.15879

**Published:** 2017-03-03

**Authors:** Jung Min Song, Kalkidan Molla, Arunkumar Anandharaj, Ingrid Cornax, M. Gerard O`Sullivan, Ameya R. Kirtane, Jayanth Panyam, Fekadu Kassie

**Affiliations:** ^1^ Masonic Cancer Center, University of Minnesota, Minneapolis, MN 55455, USA; ^2^ Department of Veterinary Population Medicine, College of Veterinary Medicine, University of Minnesota, Saint Paul, MN 55108, USA; ^3^ Department of Pharmaceutics, University of Minnesota, Minneapolis, MN 55455, USA; ^4^ Department of Veterinary Clinical Sciences, College of Veterinary Medicine, University of Minnesota, Saint Paul, MN 55108, USA

**Keywords:** triptolide, hyaluronan, hyaluronan receptor, NSCLC, orthotopic

## Abstract

Higher levels of hyaluronan (HA) and its receptors CD44 and RHAMM have been associated with poor prognosis and metastasis in NSCLC. In the current study, our goal was to define, using cellular and orthotopic lung tumor models, the role of HA-CD44/RHAMM signaling in lung carcinogenesis and to assess the potential of triptolide to block HA-CD44/RHAMM signaling and thereby suppress the development and progression of lung cancer. Triptolide reduced the viability of five non-small cell lung cancer (NSCLC) cells, the proliferation and self-renewal of pulmospheres, and levels of HA synthase 2 (HAS2), HAS3, HA, CD44, RHAMM, EGFR, Akt and ERK, but increased the cleavage of caspase 3 and PARP. Silencing of HAS2, CD44 or RHAMM induced similar effects. Addition of excess HA to the culture media completely abrogated the effects of triptolide and siRNAs targeting HAS2, CD44, or RHAMM. In an orthotopic lung cancer model in nude rats, intranasal administration of liposomal triptolide (400 μg/kg) for 8 weeks significantly reduced lung tumor growth as determined by bioluminescence imaging, lung weight measurements and gross and histopathological analysis of tumor burden. Also, triptolide suppressed expressions of Ki-67, a marker for cell proliferation, HAS2, HAS3, HA, CD44, and RHAMM in lung tumors. Overall, our results provide a strong rationale for mitigating lung cancer by targeting the HA-CD44/RHAMM signaling axis.

## INTRODUCTION

Hyaluronan (HA), a linear polysaccharide comprised of D-glucuronic acid and N-acetyl-D-glucosamine, is synthesized by three membrane-bound HA synthases, HAS1, HAS2, and HAS3 [1]. While steady-state levels of HA are generally quite low in most normal tissues, its levels dramatically increase in tumor tissues, where it enhances the proliferation, invasion, and metastasis of cancer cells [2]. High levels of HA have been correlated with poor prognosis in many different cancer types, including gastric, lung, colorectal, breast, ovarian, and bladder cancer [3–5]. Though HA signals through interaction with several cell surface receptors, the best characterized receptors in cancer cells are CD44 and RHAMM (receptor for hyaluronic acid-mediated motility). CD44, a transmembrane glycoprotein, is expressed as a wide variety of isoforms in many cells, and implicated in increased cancer cell migration, invasion, and metastasis [6–9]. It also attracted considerable interest as a cancer stem cell (CSC) marker in several malignancies of hematopoietic and epithelial origin [9]. Binding of HA to CD44 results in direct or indirect interaction of CD44 with signaling receptors, such as ErbB2, EGFR, and TGF-β, thereby influencing the activity of a variety of downstream signaling pathways, especially the MAP kinase and PI3 kinase-Akt pathways [10, 11]. Likewise, overexpression of RHAMM was reported in several cancers and HA-RHAMM signaling plays a role in neoplastic transformation and tumor progression [12–14]. In particular, lung cancer tissues showed intense RHAMM protein expression in 96% of metastatic non-small cell lung cancer (NSCLC) cases [15] and RHAMM mRNA expression was 12- and 10-fold higher in lung adenocarcinoma and squamous lung carcinoma than in matched normal lung tissues, respectively [16]. Therefore, targeting HA-CD44/RHAMM signaling axis could be a promising strategy for the prevention and treatment of lung cancer.

Triptolide, a diterpenoid triepoxide derived from the Chinese herb *Tripterygium wilfordii*, has been used for centuries to treat inflammatory and autoimmune diseases. Triptolide has also been shown to inhibit cell proliferation and induce apoptosis in a wide variety of cancer cells *in vitro* and prevent tumor growth *in vivo* via inhibition of heat shock protein (HSP) 70, c-Myc, NF-*κ*B and AP1 as well as activation of P53 [17–21]. However, the precise mechanisms of the anti-lung cancer activities of triptolide are not clear. In the present study, we first determined the role of HA-CD44/RHAMM signaling in the growth and survival of several NSCLC cells. We then examined the efficacy of triptolide to inhibit the growth of NSCLC cells *in vitro* and *in vivo* and if targeting of HA-CD44/RHAMM contributes to the growth inhibitory effects of the drug. We found that the HA-CD44/RHAMM signaling pathway plays a crucial role in the proliferation and survival of NSCLC cells and that low concentrations of triptolide significantly reduced the growth of these cells by targeting the HA-CD44/RHAMM signaling axis. Furthermore, intranasal instillation of liposomal triptolide to rats inhibited the growth of orthotopically xenografted NSCLC cells and these effects involved suppression of HA-CD44/RHAMM signaling.

## RESULTS

### Triptolide modulated the viability of lung cancer cells, and levels of cell proliferation- and apoptosis-related proteins

**Figure 1:**
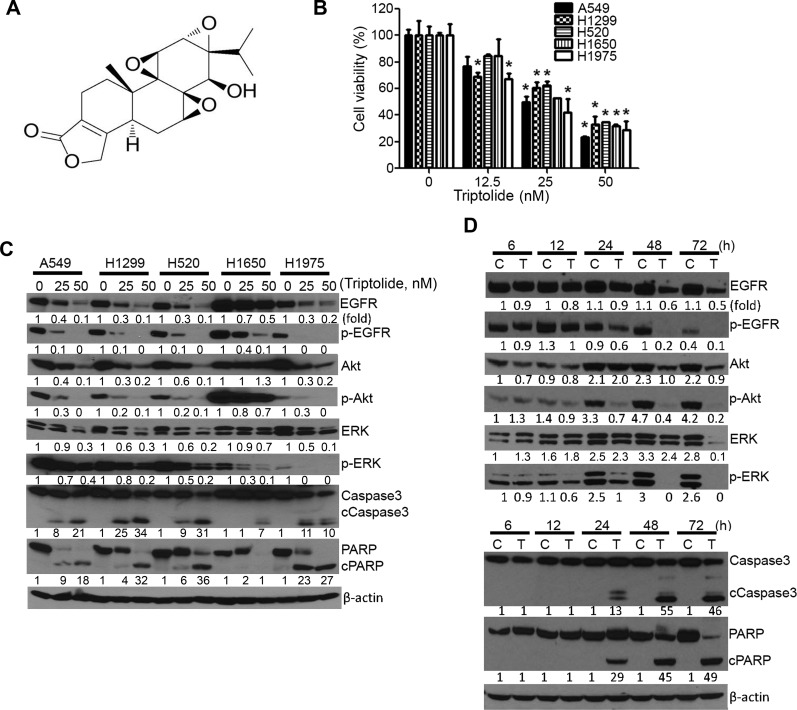
Triptolide modulated the viability of NSCLC cells and levels of cell proliferation- and apoptosis-related proteins (**A**) Chemical structure of triptolide. (**B**) Dose-dependent anti-proliferative effects of triptolide in NSCLC cells. MTT assays were performed in five NSCLC cell lines treated with DMSO or triptolide (12.5, 25 and 50 nM) for 72 h and the data were presented as percentage mean ± SD of cell viability compared to DMSO-treated cells. (**C**, **D**) Representative western immunoblotting results showing dose-dependent (C) and time-dependent (D) effects of triptolide on the expression of cell proliferation- and apoptosis-related proteins in NSCLC cells. Cells were treated with different concentrations of triptolide (0, 25 and 50 nM) for 72 h or A549 cells were treated with 50 nM of triptolide for different time periods (6, 12, 24, 48 and 72 h). Three independent assays were performed from different samples as described in materials and methods section. *P < 0.05, compared with the control group. Assays were performed in triplicate and repeated three times on different days. C, Control; T, triptolide.

NSCLC cell lines A549, H520, H1299, H1650 and H1975, harboring different genetic lesions, were exposed to triptolide at different concentrations (0, 12.5, 25 or 50 nM) for 72 h and cell viability was determined by MTT assay. As depicted in Figure 1B, the viability of all cell lines, irrespective of their molecular alterations, was reduced by triptolide in a dose-dependent manner. At the highest concentration of triptolide (50 nM), cell viability was reduced by more than 60%. Also, triptolide suppressed the colony formation ability of A549 cells in a dose-dependent manner (Supplementary Figure 1). Subsequent analysis of the dose-and time-dependent effects of triptolide on cell proliferation- and survival-related proteins showed that the drug significantly suppressed the expression of total- and phospho-EGFR, Akt and ERK and induced cleavage of caspase 3 and PARP (Figure 1C and 1D). Protein levels were modulated as early as 6 h, although significant effects were observed beginning 24 h after treatment. In line with the reduction in total protein level, the mRNA levels of Akt1 and ERK1 in A549 cells were suppressed beginning 12 h whereas EGFR mRNA was reduced at 6 h post-treatment (Supplementary Figure 2).

### Triptolide suppressed the level of HASs, HA, CD44, RHAMM, cell proliferation and survival in NSCLC cells and these effects were abrogated by exogenous HA

**Figure 2:**
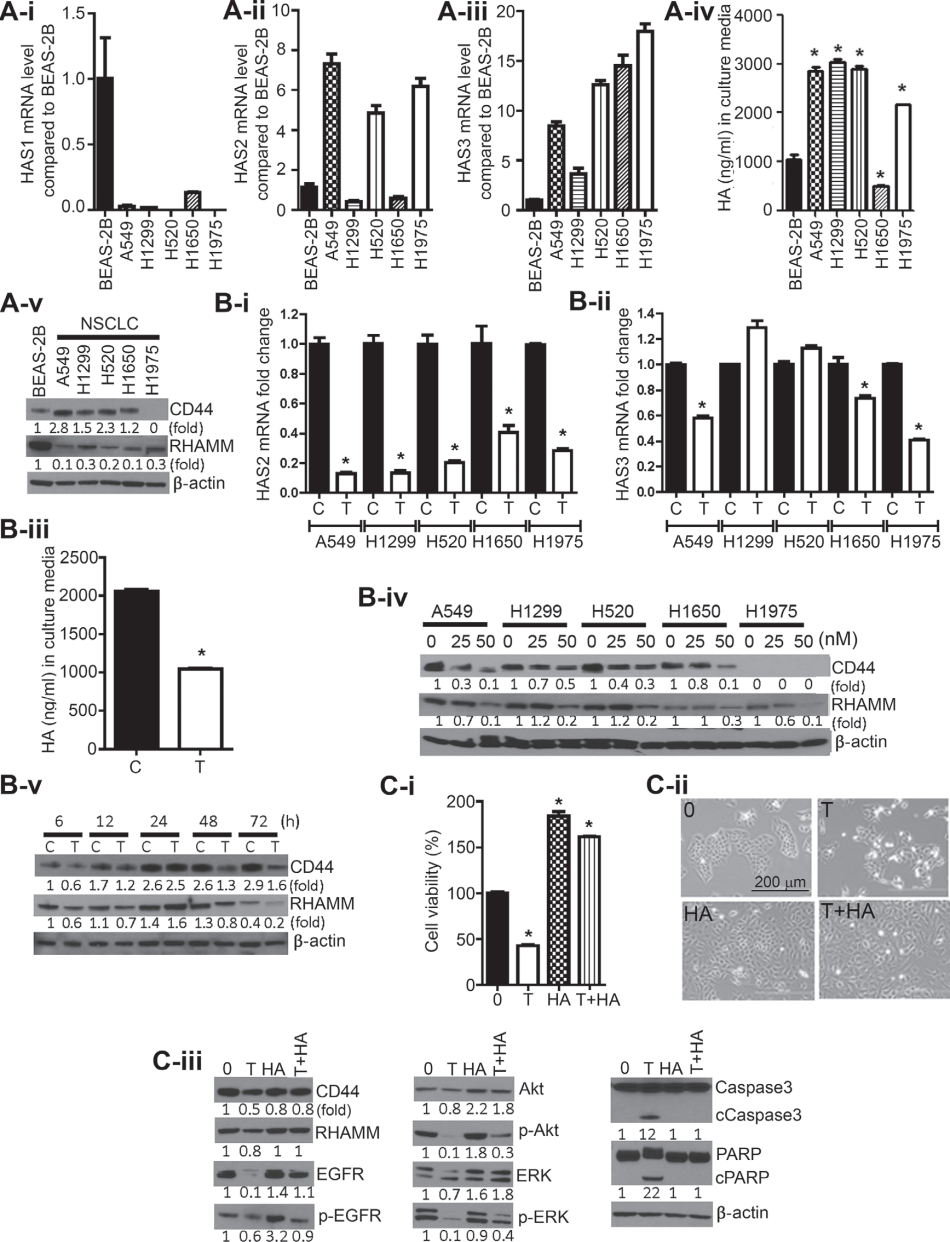
Triptolide suppressed levels of HASs, CD44, and RHAMM, and HA in NSCLC cells and exogenous HA conferred protection against the anti-proliferative and pro-apoptotic effects of triptolide (**A**) Constitutive levels of HAS1 (2A-i), HAS2 (2A-ii), HAS3 (2A-iii), HA (2A-iv) and CD44 and RHAMM (2A-v) in immortalized BEAS-2B bronchial cells and NSCLC cell lines. (**B**) Modulation of levels of HAS2 (2B-i), HAS3 (2B-ii), HA (2B-iii), CD44 and RHAMM (2B-iv; 2B-v) in NSCLC cells treated with triptolide (25 nM) or DMSO. Cells were treated with 25 nM of triptolide for 72 h with the exception of the results shown in Figure 2B-iv in which cell were exposed to 0, 25 or 50 nM of triptolide. (**C**) Exogenous HA attenuated triptolide-induced cytotoxicity and modulation of cell proliferation and survival-related proteins. C-i, A549 cells grown in RPMI media supplemented with 2.5% FBS were treated with DMSO, triptolide (25 nM), HA (2.5 mg/mL), or triptolide + HA for 72 h and cell viability determined by MTT assay. C-ii, images of A549 cells exposed to DMSO, triptolide (25 nM), HA, or triptolide + HA for 72 h. C-iii, Western immunoblotting assays showing attenuation by HA of triptolide-induced modulation in the expression of cell proliferation and survival-related proteins. *P < 0.05. Assays were performed in triplicate and repeated three times on different days. C, Control; T, triptolide.

First, we compared basal mRNA levels of the three HAS isoforms (HAS1, HAS2 and HAS3), CD44 and RHAMM in immortalized BEAS-2B bronchial cells and NSCLC cell lines. Compared to that in BEAS-2B cells, the expression of HAS1 was lower in all NSCLC cell lines (Figure 2A–i), whereas HAS2 (Figure 2A-ii) and HAS3 (Figure 2A-iii) were overexpressed in most of the cell lines. Consistent with these results, measurement of HA accumulation in the culture media showed that A549, H1299, H520 and H1975 cells secreted a 2–3 fold higher level of HA as compared to the amount secreted by BEAS-2B cells (Figure 2A-iv); the level of HA in H1650 cells was lower than that of BEAS-2B cells. CD44 and RHAMM were expressed at gene (data not shown) and protein levels in all cell lines with the exception of H1975 cells which do not express CD44 (Figure 2A-v). The level of CD44 was higher in NSCLC cells as compared to the level in BEAS-2B cells, whereas RHAMM expression was higher in BEAS-2B cells than in NSCLC cells.

To assess if triptolide suppresses HA synthesis in lung cancer cells, NSCLC cells were treated with the drug (25 nM) for 72 h and expressions of HAS2 and HAS3 were determined. Triptolide significantly suppressed HAS2 expression in all cell lines by at least 60% (Figure 2B-i), whereas the effect on HAS3 was relatively weaker (Figure 2B-ii). Subsequent analyses of time-dependent reduction in the expression of these genes showed that triptolide suppressed mRNA level of HAS2 and HAS3 beginning at 6 h and 12 h, respectively (Supplementary Figure 2). In line with the suppression of HAS2 and HAS3, triptolide-treated A549 cells secreted a lower amount of HA (2-fold lower) in the culture media compared to DMSO-treated cells (Figure 2B-iii). Analysis of HA levels at different time points indicated significant suppression as early as 12 h post-treatment (Supplementary Figure 3).

Similarly, treatment of NSCLC cells with triptolide (0, 25 or 50 nM) reduced the expression of CD44 and RHAMM at both gene (data not shown) and protein levels (Figure 2B-iv). Analysis of the time-dependent effects of triptolide on CD44 and RHAMM expression showed that levels of the two proteins were suppressed as early as 6 h after treatment, increased at 24 h and reduced again after 48 h (Figure 2B-v).

To determine if the anti-proliferative and pro-apoptotic effects of triptolide are mediated via inhibition of HA synthesis, A549 cells were treated with DMSO, triptolide (25 nM), HA (2.5 mg/mL) or triptolide + HA and cell viability and modulation of cell proliferation-and survival-related proteins were determined. Compared to viability of DMSO-treated cells, the viability of triptolide-treated cells decreased by 60%, whereas that of HA-treated cells increased by two-fold (Figure 2C-i), However, upon co-exposure of the cells to triptolide and HA, triptolide-induced cytotoxicity was completely abrogated (cell viability was 1.8-fold higher than that of control cells. Images of A549 cells treated with DMSO, triptolide, HA or triptolide + HA are shown in Figure 2C-ii. Consistent with results in MTT assays, triptolide reduced levels of CD44, RHAMM, EGFR, ERK and Akt and activated caspase 3 and PARP cleavage (Figure 2C-iii). On the other hand, co-treatment with triptolide and HA markedly attenuated the effect of triptolide on these proteins.

### Silencing of HAS2, CD44 or RHAMM reduced NSCLC cell viability and suppressed the level of cell proliferation- and apoptosis-related proteins

**Figure 3:**
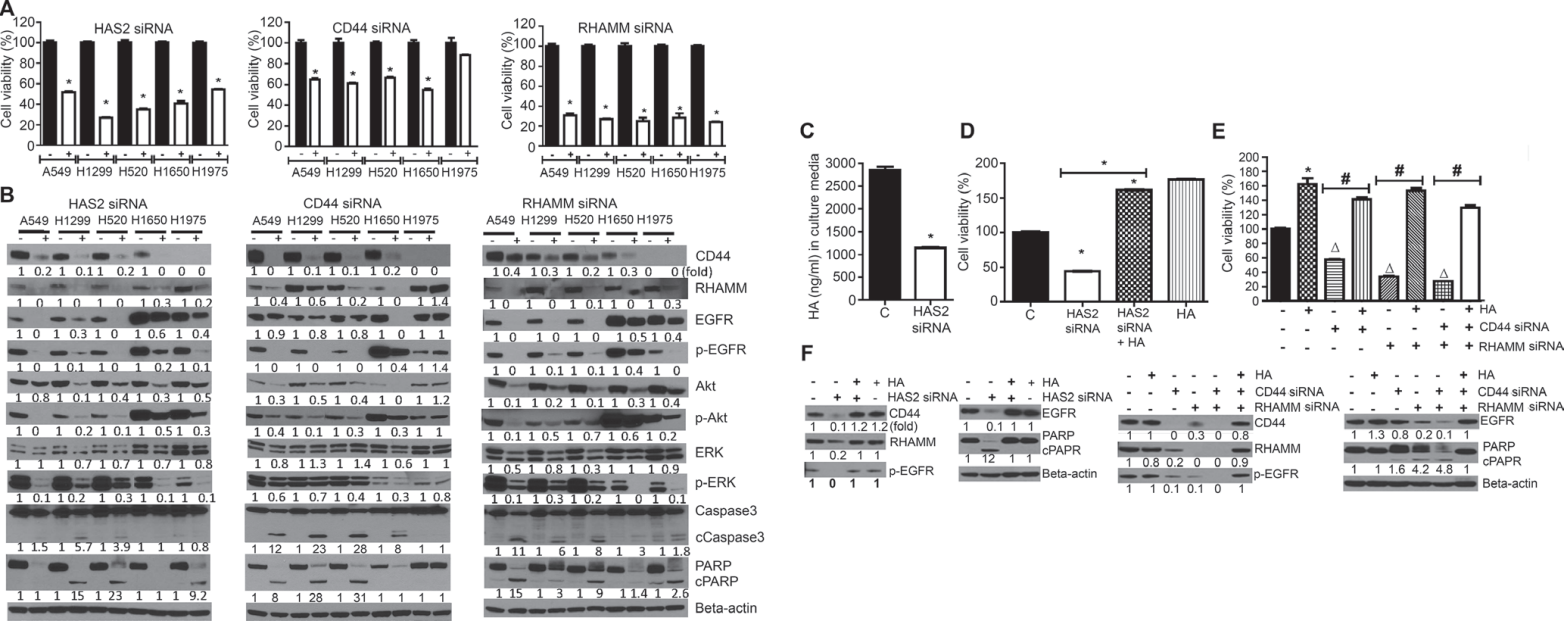
siRNA-mediated silencing of HAS2, CD44 or RHAMM reduced NSCLC cell viability and the level of cell proliferation- and apoptosis-related proteins and these effects were modulated by exogenous HA (**A**) Effects of HAS2, CD44 and RHAMM siRNA on the viability of NSCLC cells. Each cell line was transfected with the individual siRNAs and cell viability was determined by MTT assay as described in the Materials and Methods section. (**B**) HAS2, CD44 and RHAMM siRNAs modulated the expression of cell proliferation-and survival-related proteins. NSCLC cells were transfected with the siRNAs and Western immunoblotting was performed as described in the Materials and Methods section. (**C**) Effect of HAS2 siRNA on HA synthesis. A549 cells were transfected with HAS2 siRNA and accumulation of HA in the culture media was determined as described in the Materials and Methods section. (**D**, **E**) Exogenous HA (2.5 mg/mL) rescued cells from the cytotoxic effects of siRNAs targeting HAS2 (D), CD44, RHAMM or CD44 + RHAMM (E). A549 cells were treated with HAS2, CD44, RHAMM or CD44 + RHAMM siRNAs or HA alone or siRNA + HA and cell viability determined by MTT assay. (**F**) Exogenous HA attenuated the effects of triptolide on cell proliferation-and apoptosis-related proteins. A549 cells grown in RPMI media supplemented with 2.5% FBS were treated with HAS2, CD44, RHAMM or CD44 + RHAMM siRNAs or HA alone or siRNA + HA and expression of the proteins determined by Western immunoblotting. For all experiments, at least three independent assays were carried out. *P < 0.05, compared to treatment with DMSO-treated cells; Δ, compared to DMSO-treated cells; ♯, compared to treatment with siRNA only.

In order to further assess the role of the HA-CD44/RHAMM signaling in the growth and survival of NSCLC cells, we transfected the cells with HAS2, CD44 or RHAMM specific siRNAs or scrambled siRNA and effects on cell viability and expression of cell proliferation- and apoptosis-related proteins were determined. Transfection of NSCLC cells with HAS2, CD44, or RHAMM siRNA significantly reduced the expression of the respective genes (data not shown) and reduced the viability of NSCLC cells (Figure 3A), albeit with different efficiency. RHAMM siRNA was the most efficient (about 70% reduction), followed by HAS2 siRNA (50–75% reduction) while CD44 siRNA caused relatively weaker cytotoxic effects (20–40% reduction). In line with the reduction in cell viability, all five NSCLC cell lines transfected with HAS2, or RHAMM siRNA and four cell lines transfected with CD44 exhibited suppression of CD44, RHAMM, and total- and phosphorylated-EGFR, Akt and ERK, whereas the pro-apoptotic proteins caspase 3 and PARP were activated in most of the cell lines as evidenced by enhanced cleavage (Figure 3B). Again, the efficacy of RHAMM siRNA was the strongest. Subsequently, we examined if HA rescues A549 cells from the cytotoxicity of HAS2, CD44 and RHAMM siRNAs. As shown in Figure 3C, silencing of HAS2 reduced accumulation of HA in the culture media by 60%. Consistent with the reduction in HA synthesis, the viability of A549 cells was reduced by 56%, whereas concurrent exposure of the cells to HAS2 siRNA and HA (2.5 mg/mL) not only completely rescued the cells from the cytotoxic effects of HAS2 silencing, but also increased cell viability by 1.6-fold (Figure 3D). As expected, exposure of the cells to HA alone increased cell viability (1.7-fold). Similar to the results observed with HAS2 siRNA, siRNAs targeting CD44, RHAMM and CD44 + RHAMM reduced the viability of A549 cells by 43, 66, and 73%, while co-treatment with HA and siRNAs targeting CD44, RHAMM or CD44 + RHAMM increased cell viability by 1.4-, 1.5- and 1.3-fold, respectively, compared to the cell viability in control cells (Figure 3E). In line with the cell viability results, silencing of HAS2, CD44 and RHAMM in A549 cells suppressed the level of CD44, RHAMM, EGFR and phospho- EGFR, albeit to different degrees, and caused PARP cleavage and these effects were reversed upon co-treatment with HA and the siRNAs (Figure 3F).

### Triptolide and CD44 siRNA reduced the proliferation and self-renewal of putative lung cancer stem cells

**Figure 4:**
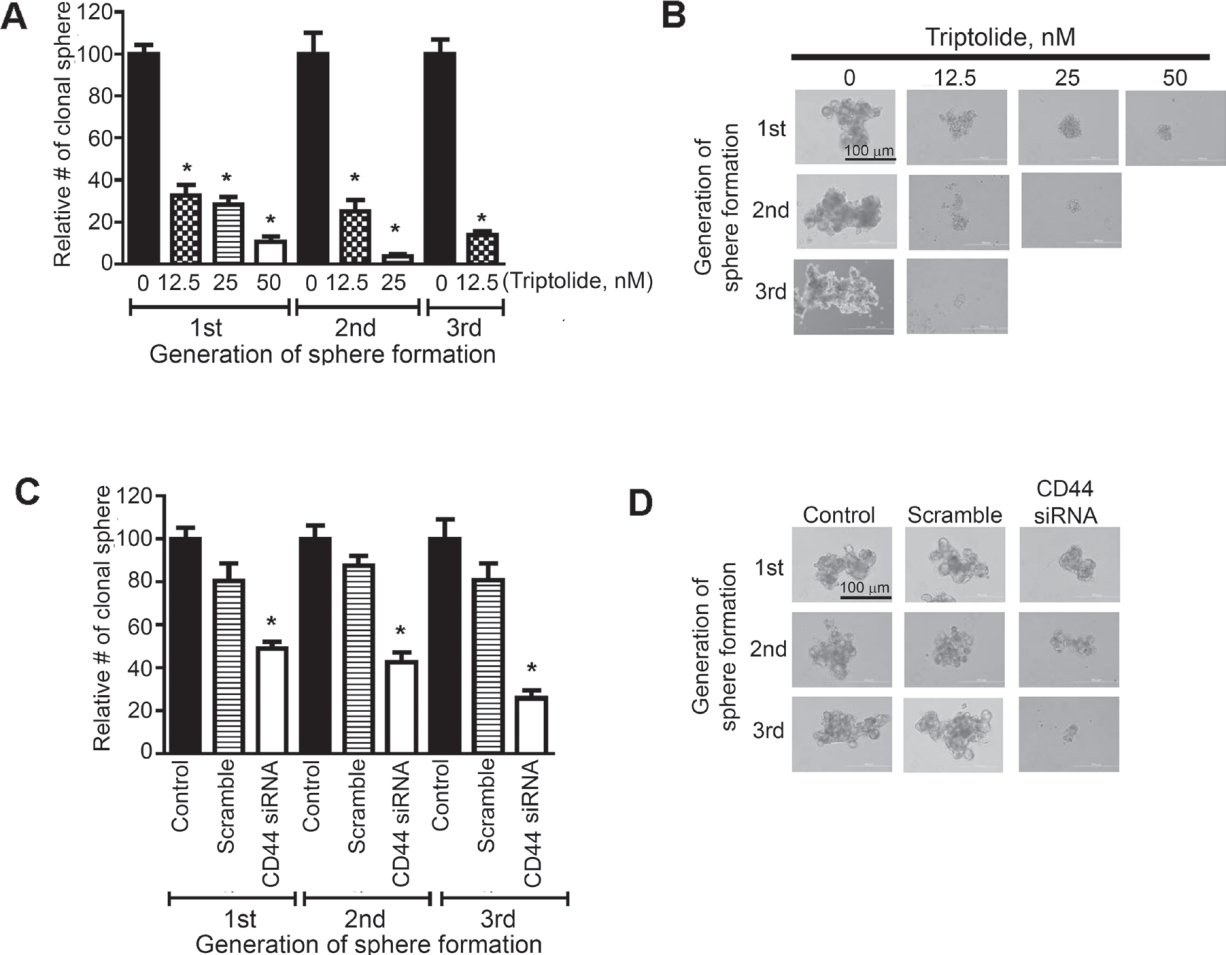
Triptolide and CD44 siRNA reduced the proliferation and self-renewal of putative lung cancer stem cells expressing CD44 (**A**, **C**) Effects of triptolide (A) or CD44 siRNA (C) on the number of primary-, secondary and tertiary-pulmospheres generated from A549 cells. Cells were treated with triptolide (12.5, 25 and 50 nM) or CD44 siRNA and the frequency of pulmospheres determined as described in the Materials and Methods section. (**B**, **D**) Images of primary, secondary and tertiary pulmospheres generated from triptolide (B)- or CD44 siRNA (D)- treated A549 cells. At least three independent assays were carried for these assays. *P < 0.05.

Triptolide has been shown to suppress the growth of cancer cells expressing the cancer stem cell markers CD133 [22] and ALDH1 [23]. Since triptolide suppressed the expression of CD44, another marker for cancer stem cells, we sought to determine, using pulmosphere assay, if the drug inhibits the proliferation and self-renewal of putative lung cancer stem cells. Triptolide dose-dependently reduced not only the number of primary pulmospheres generated from A549 cells (Figure 4A), but also the size of the pulmospheres (data not shown). Furthermore, upon subsequent passage of triptolide-treated primary or secondary pulmospheres, the number of secondary and tertiary pulmospheres, respectively, was significantly reduced. Images of primary, secondary and tertiary pulmospheres generated from DMSO- or triptolide-treated A549 cells are depicted in Figure 4B. Similarly, transfection of A549 cells with CD44 siRNA significantly reduced the number of primary, secondary and tertiary pulmospheres (Figure 4C) and images of these spheres are shown in Figure 4D.

### Liposomal triptolide significantly reduced orthotopic growth of A549 cells in rats

**Figure 5:**
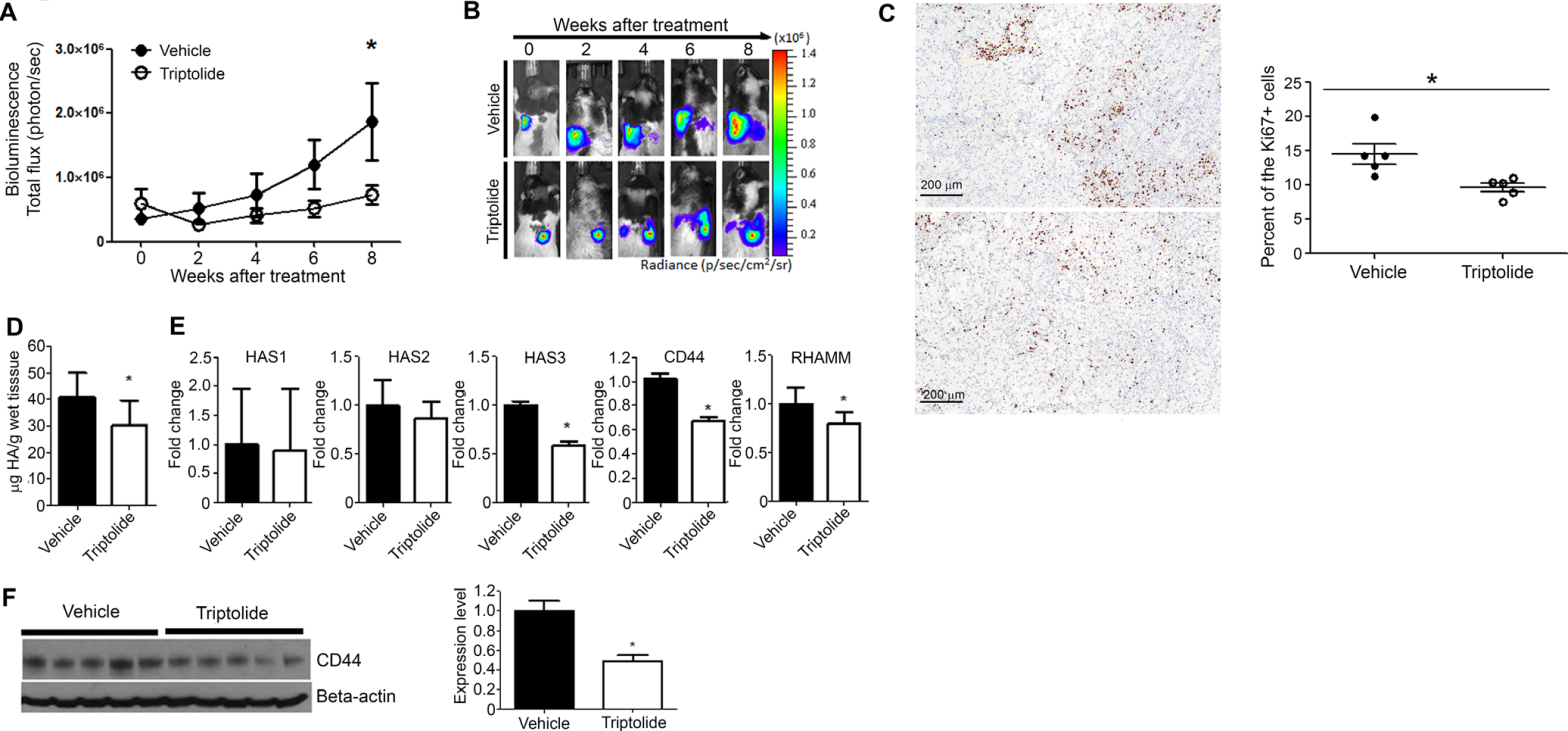
Liposomal triptolide significantly reduced the growth of orthotopic lung tumor in nude rats (**A**) Effects of triptolide on the growth of orthotopically transplanted A549 cells as determined by bioluminescence imaging. Rats in which luciferase expressing A549 cells were implanted in the lung were given liposome-encapsulated triptolide (400 μg/kg) and the growth of lung tumors monitored by bioluminescence imaging as described in the Materials and Methods section. (**B**) Representative results of bioluminescence imaging studies in the vehicle and triptolide groups. (**C**) Representative images of Ki-67 expression in the lung tissues of vehicle- (top panel) or triptolide-treated (bottom panel) rats. Right panel: Bar graph showing the percent of Ki-67-positive lung tumor cells (mean ± SD) in vehicle- or triptolide-treated rats. (**D**) Levels of HA in lung tumor tissues of vehicle- and triptolide-treated rats. HA levels were measured by HA ELISA-like assay kit as described in the Materials and Methods section. (**E**) Levels of HAS1, HAS2, HAS3, CD44, and RHAMM mRNA transcripts in lung tumor tissues of vehicle- and triptolide-treated rats. (**F**) Left panel: Representative results showing Western immunoblotting analyses of CD44 expression in lung tumor tissues of vehicle- and triptolide-treated rats. Right panel: Quantification of CD44 expression in lung tumor tissues. The results from Figure 5D–5F were obtained from at least three independent assays performed on different days. *P < 0.05.

Biweekly bioluminescence imaging showed that signal intensity was significantly lower in the triptolide group, in particular during the later time points, as compared to the level in the vehicle group, indicating a reduction in the growth of lung tumors (Figure 5A). Representative results are depicted in Figure 5B. In line with the reduction in bioluminescence signal, rats treated with triptolide had significantly lower lung weight compared to the vehicle group (2.98 g versus 4.49 g, Table 1), but body and liver weights of the rats in the two groups were similar. Also, analysis of tumor burden indicated that triptolide significantly reduced tumor burden (2.38 in the triptolide group versus 3.08 in the vehicle group, Table 1).

**Table 1:** Effect of liposomal triptolide on the rat body weight, the wet lung weight and gross tumor burden in the lung

Treatment group	*N*	Body weight, g	Liver weight, g	Lung weight, g	Tumor burden (0-4, arbitrary size)
Control	5	315.5 ± 29.05	11.04 ± 1.40	2.10 ± 0.66	0
Vehicle	12	290.88 ± 26.16	9.70 ± 0.88	4.49 ± 0.94	3.08 ± 0.90
Triptolide	13	292.40 ± 19.99	9.86 ± 0.95	2.98 ± 0.68*	2.38 ± 0.77*

* *P* < 0.05, compared to vehicle.

To determine if triptolide reduced the rate of cell proliferation in lung tumor tissues, expression of Ki-67, a marker of proliferation associated nuclear antigen expressed in replicating cells during all phases of the cell cycle [24], was compared between the vehicle and triptolide groups. These studies showed that the frequency of Ki-67-positive cells was significantly (*P* = 0.03) lower in the triptolide group compared to the vehicle group (9.6 vs 14.5%, respectively, Figure 5C). Moreover, to determine if triptolide modulated HA-CD44/RHAMM signaling in lung tumor tissues, we analyzed HA accumulation and mRNA levels of HAS1, HAS2, HAS3, CD44 and RHAMM in lung tumors from control and triptolide-treated rats. HA level in the tumor tissue was significantly reduced by triptolide (Figure 5D). In line with this, level of HAS2 was downregulated although the change was not significant, whereas HAS3 expression was significantly reduced (Figure 5E). Also, mRNA levels of both CD44 and RHAMM and protein levels of CD44 were suppressed by triptolide (Figure 5E and 5F).

## DISCUSSION

In the current study, we showed that the HA-CD44/RHAMM signaling axis plays a crucial role in the proliferation and survival of NSCLC cells. Furthermore, the phytochemical triptolide inhibited the proliferation and survival of NSCLC cells having different genetic alterations, at least in part, by targeting members of the HA-CD44/RHAMM signaling pathway, thereby suppressing the expression and activation of downstream effector proteins, including EGFR, Akt and ERK and increasing the activation of pro-apoptotic proteins caspase 3 and PARP. In a rat model of orthotopic lung cancer, intranasal administration of liposomal triptolide significantly inhibited lung tumor growth and these effects were paralleled by reduced tumor cell proliferation, and lower HAS, HA and CD44/RHAMM levels in the tumor tissues.

Although triptolide has been shown to have anticancer effects in several preclinical cancer models, unfavorable toxicity profile of the drug [25] has hindered its clinical development as a cancer therapeutic agent. To overcome this problem, in the present study triptolide was formulated in liposomes, small lipid vesicles with a composition close to lung surfactant, and administered at low concentrations, via intranasal instillation. As a result, liposomal triptolide did not induce any adverse effects as evidenced by absence of body weight loss or histopathological changes in the liver (data not shown). Liposomal formulation of anti-cancer drugs has been consistently shown to enhance drug efficacy while reducing toxicity. For instance, liposome-encapsulated cisplatin and paclitaxel, chemotherapeutic drugs used widely for the treatment of NSCLC, led to delivery of higher drug dosage at the tumor site and reduced non-target toxicity and drug resistance [26], which could be attributed to altered pharmacokinetics and bio-distribution of the drugs [27]. Moreover, intranasal instillation of triptolide would also increase the therapeutic effectiveness of the drug by enabling delivery of high concentration of the drug at the target site and averting barriers to therapeutic efficacy such as poor gastrointestinal absorption and first-pass metabolism in the liver, while reducing systemic toxicity by diminishing the concentration of the drug at non-target tissues [28].

NSCLC tumors have been shown to exhibit high levels of HA [29, 30] as well as its receptors CD44 and RHAMM [15, 16, 30] and overexpression of these molecules was associated with poor prognosis and metastasis of NSCLC [15, 30]. In an effort to identify downstream effectors of HA-CD44/RHAMM pathway, we silenced the expression of HAS2, CD44 or RHAMM, and determined potential modulation of proteins involved in cell proliferation and survival. We observed that silencing HAS2, CD44 or RHAMM genes suppressed the expression and activation of EGFR, Akt, and ERK and activated the pro-apoptotic proteins caspase 3 and PARP, indicating that the interaction between HA and CD44/RHAMM regulate the EGFR/Akt/ERK pathway and thereby enhancing cell proliferation and survival. These results are consistent with previous reports in malignant colon, prostate, and breast carcinoma cells, in which HA-CD44 interaction activated multiple RTKs including EGFR as well as assembly of lipid-raft-integrated signaling complexes containing activated RTKs, CD44, ezrin, PI3-kinase (PI3K) and the chaperone molecules HSP90 and CDC37 [31]. Likewise, HA-RHAMM interaction enhanced phosphorylation of EGFR, ERK1/2, and STAT3, and these effects were blocked by gefitinib, an inhibitor of EGFR signaling, suggesting that HA-RHAMM signaling uses EGFR to promote tumor cell survival [32]. Exogenous HA completely overcame the combined silencing of CD44 and RHAMM, which suggests that HA could bind to other receptors. Indeed, HA has been shown to promote the proliferation and migration of cancer cells by interacting with TLR4 and knockdown of TLR4 inhibited these effects of HA [33].

Our studies indicated that HA acts not only as a ligand of CD44 and RHAMM, but also modulates their expression as HAS2 siRNA downregulated CD44 and RHAMM. These effects could be responsible, at least in part, for the rescue by HA of HAS2, CD44 and RHAMM siRNA-induced cytotoxicity in A549 cells. Similar observations were made in bladder cancer cells in which silencing of HAS1 suppressed the level of CD44 variant isoforms. In the same study, exogenous HA increased CD44 levels which were responsible for the partial rescue of the phenotype of the cells, but these effects were lost when CD44 expression was blocked [34]. In the present study, CD44 siRNA silenced not only CD44 expression but also that of RHAMM and the converse was true for RHAMM siRNA. Veiseh and colleagues [35] also observed a dual role of RHAMM as an HA receptor and a regulator of CD44 expression in RHAMM-transfected mesenchymal progenitor cells. At present, it is unclear how CD44 and RHAMM regulate each other. One possibility is that CD44 and RHAMM promote each other`s transcription or mRNA stability. Another issue that needs further investigations is the relative roles of standard (CD44s) and variant (CD44v) isoforms of CD44 in cancer development and progression. Assessment of the expression of CD44 in 12 human lung cancer cell lines and 23 paraffin-embedded lung cancers showed that CD44s is the predominant isoform [36]. In line with this, among 6 lung cell lines used in the present study, H1650 expressed both CD44s and CD44v, whereas BEAS-2B, A549, H1299 and H520 cells expressed only CD44s; H1975 cells expressed neither of the CD44 isoforms. It also appears that the isoform of CD44 depends on the type of cancer. For instance, acute myeloid leukemia (AML) and Glioblastoma express solely CD44s, whereas in colorectal cancer more than 75% of patients express CD44v8-v10 as the predominant isoform [37]. The CD44 isoform status has also been shown to predict treatment response to RG7356, a humanized monoclonal antibody directed against the constant region of CD44. In a study involving 37 tumor xenografts, models using cell lines expressing predominantly CD44s were found to be responsive to RG7356 treatment (8/19, 42%), whereas none of the models with predominant CD44v tumor cells responded to RG7356 [37].

The HA-CD44/RHAMM signaling pathway is an attractive target for cancer prevention and therapy as it regulates critical steps in tumor growth and progression, including cell proliferation, survival, migration, invasion, and the proliferation and self-renewal of cancer stem cells. Small-molecule inhibitors, antibodies and vaccines have been developed to target the different HA family members [38]. However, with the exception of some anti-CD44 antibodies, which were found to be highly toxic, none of the agents have so far been tested in cancer patients. In the present study, we showed that triptolide, an anti-cancer agent found to be effective against a variety of tumors in preclinical models [17–19] and now being tested in a Phase I clinical trial (https://clinicaltrials.gov/ct2/show/NCT01927965), is a potent inhibitor of HA-CD44/RHAMM signaling and associated oncogenes, including EGFR, Akt and ERK. Interestingly, triptolide simultaneously targeted different members of HA signaling, including HAS2, HAS3, CD44 and RHAMM. Co-treatment of NSCLC cells with exogenous HA and triptolide completely abrogated triptolide-induced cytotoxicity and modulation of CD44, RHAMM, EGFR, Akt, ERK, caspase 3 and PARP levels, suggesting that targeting of HA-CD44/RHAMM signaling is the principal mechanism through which triptolide suppresses the proliferation and survival of NSCLC cells. Although this is the first detailed report on the inhibition of HA-CD44/RHAMM signaling by triptolide, previous studies have shown that triptolide inhibited HA synthesis in the extracellular matrix and thereby enhanced drug delivery to mouse pancreatic tumors [39]. Triptolide-induced suppression of HA synthesis was ascribed to inhibition of the transcriptional activity of Sp1, which is instrumental in the regulation of HA synthase genes [40, 41]. Triptolide also suppressed interferon-gamma-induced HA synthesis in fibroblasts [42]. Heat shock proteins were reported to be critical molecular targets of triptolide in some cancer models [43, 44]. However, in the current study, triptolide suppressed the expression of heat shock proteins only at concentrations ≥ 50 nM (data not shown), indicating that heat shock proteins are not the primary target of triptolide in NSCLC cells.

Triptolide also inhibited the proliferation and self-renewal of spheres in pulmosphere assays, which have been widely used to retrospectively identify putative lung cancer stem cells with increased tumor-propagating ability *in vivo* [45]. These results are consistent with triptolide-induced downregulation of CD44, which is considered to be a compelling marker for cancer stem cells of many solid malignancies [46]. However, the relationship between CD44 expression and the stemness of cancer cells is unclear. One potential missing link might be overexpression of EGFR upon activation of HA-CD44 signaling. EGFR has been shown to regulate the expression of Sox2, a transcription factor that maintains the self-renewal of embryonic stem cells, via EGFR-Src-Akt signaling in NSCLCs resulting in the modulation of self-renewal and expansion of stem-like cells [47]. Abrogation of EGFR signaling through pharmacological or genetic inhibitors suppressed the self-renewal growth and expansion of cancer stem cells. These results indicate that targeting HA-CD44-mediated enhancement of EGFR expression and activation in cancer stem cells could be a promising approach to suppress the progression of lung cancer. Triptolide could also be used in conjunction with EGFR TKIs as long-term therapy with these drugs was shown to enrich cancer stem cells [48, 49].

In conclusion, the present report demonstrated that HA-CD44/RHAMM signaling plays a pivotal role in the proliferation and survival of NSCLC cells and provides a strong rationale for the clinical use of triptolide to mitigate lung cancer exhibiting activation of HA-CD44/RHAMM signaling axis.

## MATERIALS AND METHODS

### Cells and reagents

A549 cells (K-Ras mutant, adenocarcinoma) stably expressing the luciferase gene were from Caliper Life Sciences (Waltham, MA). H1650 (EGFR tyrosine kinase domain ΔE746-A750, exon 19 mutant, adenocarcinoma) and H1975 (L858R kinase domain mutation in exon 21 and a second mutation at T790M, P53 mutation, adenocarcinoma) cell lines were obtained from Dr. Shujun Liu (Hormel Institute, University of Minnesota). H520 (FGFR amplification, low mRNA level of P53, squamous cell carcinoma) and H1299 (P53 deletion, adenocarcinoma) cell lines were purchased from ATCC. BEAS-2B immortalized bronchial cells were kindly provided by Dr. Klein-Szanto (Fox Chase Cancer Center, Philadelphia, PA). Upon receiving the cell lines, the authenticity of the cells was determined by short tandem repeat analysis technology at MD Anderson`s Cell Line Core Facility. We routinely carry out mycoplasma screening for all of our cell lines. All NSCLC cell lines used in this study were cultured in RPMI 1640 medium supplemented with 10% FBS in 5% CO_2_ incubator at 37°C, whereas BEAS-2B cells were maintained in keratinocyte serum-free medium with recommended supplements (Life Technologies, Gaithersburg, MD) under the same condition. Triptolide (chemical structure shown in Figure 1A) was purchased from Cayman chemicals (Ann Arbor, MI). Anti-CD44, anti-phospho-Akt (Ser 478), anti-total Akt, anti-phospho-ERK (T202/Y204), anti-total ERK, anti-phospho-EGFR (Y1068), anti-total EGFR, anti-caspase 3, anti-β-actin and goat anti-rabbit IgG secondary antibody were from Cell Signaling Technology (Beverly, MA). Anti-poly (ADP-ribose) polymerase (PARP) was obtained from Santa Cruz Biotechnology. Anti-RHAMM (CD168) antibody was purchased from Abcam (Cambridge, MA). Hyaluronic acid sodium salt (HA, 500 kDa) was purchased from Lifecore Biomedical (Chaska, MN).

### Cell viability assay

NSCLC cells were plated on a 24-well plate at a density of 20,000 cells/well, grown in culture media containing 10% FBS for 24 h and exposed to triptolide (0–50 nM) for 72 h followed by methylthiazoletetrazolium (MTT, Biotium, Hayward, CA) treatment (40 μL per well) for 4 h. For assays with siRNAs, cells were transfected with HAS2, CD44 or RHAMM siRNAs (100, 200 or 50 nM, respectively, Valencia, CA) in lipofectamin RNAiMAX reagent (Invitrogen, Carlsbad, CA) for 6 h. In some experiments, A549 cells were co-treated with HA (2.5 mg/mL) and triptolide or HAS2, CD44 or RHAMM siRNAs and grown in media containing 2.5% FBS.

### Colony formation assay

Cells treated with DMSO or triptolide (12.5 and 25 nM) for 24 h were seeded in 60 mm culture plates and incubated for two weeks in RPMI 1640 medium supplemented with 10% FBS in the absence of triptolide. The colonies were stained for 10 min at room temperature with 0.5% crystal violet prepared in 30% ethanol. After washing out the dye with tap water, colonies were counted. *P < 0.05, compared with the untreated A549 cells. Assays were performed in three times on different days.

### Pulmosphere formation assay

To study the effect of triptolide on stem-cell-like properties of lung cancer cells, sphere formation assays were performed as described previously [50]. Briefly, A549 cells were plated in six-well ultra-low attachment plates (Corning, Acton, MA) at a density of 3,000 cells/mL in DMEM-F12 culture media (Corning, Manassas, VA) supplemented with 20 ng/mL EGF, 20 ng/mL bFGF, 0.4% BSA, 1x Insulin-Transferrin-Selenium solution, 1 mM HEPES buffer, and 1% Penicillin/Streptomycin (Gibco, Grand Island, NY). After 7 days of culture with and without triptolide (0–50 nM), the number of clonal spheroids was analyzed. For the second and third generation of clonal sphere culture, primary spheroids or secondary spheroids, respectively, were dissociated using Accutase (Innovative cell technology, San Diego, CA). Then, 500 cells per well were re-seeded in 24-well ultra-low attachment plates, in the absence of triptolide, and incubated for 7 days.

### Measurement of HA levels in culture media or lung tumor tissue

For the assay with culture media, we used media in which cells were treated with DMSO, triptolide (25 nM) or HAS2 siRNA (100 nM) for 72 h. For the assay with lung tumor tissues, frozen tumor tissues (10 mg) were homogenized in 1.0 mL of 150 mM Tris–HCl (pH 8.3), 150 mM sodium chloride, 150 mM calcium chloride and proteinase K (1 mg/mL), the samples digested overnight at 55°C and the homogenates clarified by centrifugation at 4°C (18,000g for 5 min). Supernatants were collected and placed in a boiling bath for 10 min to inactivate any proteases. The amount of HA in the samples was determined using a quantitative HA test kit (Corgenix, Broomfield, CO), which detects HA based upon the binding of HA to a labeled HA binding protein. The absorbance of samples and HA standards was read at 450 nm on a microplate reader (Synergy HT, Biotek Instruments, Winooski, VT). The concentration of HA in each sample was determined by interpolation from a standard curve ranging from 0 to 800 ng/mL.

### Preparation and characterization of liposomal triptolide

Liposomal encapsulation of triptolide was carried out as we described previously [51] with slight modifications. Briefly, the mixture of 1,2-dipalmitoyl-sn-glycerol-3-phosphatidylcholine (DPPC), cholesterol, 1,2-distearoyl-sn-glycerol-3-phosphatidylethanolamine (DSPE) and triptolide in weight ratios of 1:0.16:0.25:0.11 were dissolved in 5 mL chloroform/methanol at a ratio of 3:1 (v/v). The mixture was gently warmed to 40°C in a round-bottomed flask, and the solvent was evaporated under vacuum in a rotary evaporator until a thin lipid film was formed. The dried lipid films were left overnight and sonicated in 5% glucose solution followed by concentration and lyophilization. Blank liposomes were synthesized by the same method without the addition of triptolide. Hydrodynamic diameter of triptolide liposomes was measured using a Delsa Nano C particle size analyzer (Beckman Coulter, Fullerton, CA) and was found to be ~240 ± 67 nm. HPLC analysis of the liposome encapsulated triptolide showed ~77 ± 5% encapsulation efficiency.

### Assessment of the lung tumor growth inhibitory effects of liposomal triptolide in an orthotopic rat model of lung cancer

An orthotopic rat model of lung cancer was established as described previously [52]. Briefly, male nude rats (Cr:NIH-rnu, 8–10 weeks age and weighing about 200 g) were obtained from the National Cancer Institute, Frederick Cancer Research Facility. All studies were approved by the Institutional Animal Care and Use Committee of the University of Minnesota. After 24 h, rats were anesthetized with 1.5% of isoflurane and a total 20 × 10^6^ luciferase expressing A549 cells (in 0.5 ml of RPMI 1640 media supplemented with 10% FBS and 7 μmol/L EDTA) were inoculated into the lung via intranasal instillation. Rats were observed until fully recovered from the effects of the procedure. Rats in the negative control group were instilled with RPMI 1640 culture media only. Bioluminescence imaging was performed biweekly to monitor the growth of lung tumors as follows. Rats were anesthetized with 1.5% of isoflurane for 2 min, injected with D-luciferin (ip, 100 mg/kg in 1000 μL 0.9% saline solution), and imaging performed using the IVIS spectrum *In Vivo* imaging system (Xenogen, Alameda, CA). Quantitative analysis was performed by defining a standard area over the mid-lung zone and determining total flux level (p/sec) over the area of interest.

Four weeks following inoculation of the tumor cells, rats with similar level of bioluminescence were assigned to the triptolide (*n* = 13) or vehicle groups (*n* = 12) groups and treated, by intranasal instillation, with liposome encapsulated triptolide (400 μg/kg in 500 μL of 5% aqueous dextrose solution) or the same volume of empty liposome, respectively, for eight weeks (five times a week, a total 40 treatments). Bioluminescence signals were measured biweekly throughout the study. Upon termination of the study, rats were euthanized by an overdose of carbon dioxide, the lungs harvested and the lung tumor burden determined by measuring the wet weights of the lungs and subjectively assigning scores of 1–4 to the surface tumors and palpable parenchymal tumors; scores of 1–4 represent minimal, mild, moderate or marked tumor burden, respectively.

### Histopathological and immunohistochemistry (IHC) analysis of lung tumor tissues

For IHC preparations, 4 μm formalin-fixed, paraffin-embedded sections of tissue were deparaffinized and rehydrated, followed by antigen retrieval in a steamer prior to IHC procedures performed on a Dako Autostainer. IHC for Ki-67 was performed using a mouse monoclonal antibody (Clone MIB-1 from Dako) as primary antibody. Detection of bound primary antibody was achieved using the Dako EnVision + System-HRP kit, with DAB as the chromogen. The percentage of Ki-67-positive nuclei in lung tumor cells of control and triptolide-treated rats (5 rats/group) was determined using the Image J program.

### Quantitative reverse transcription–PCR analysis

Total RNA was extracted by using the miRNeasy Mini Kit (Qiagen, Valencia, CA) according to the manufacturer's instruction. The purity and integrity of total RNA were confirmed by Nanodrop. The first-strand complementary DNA was synthesized and quantitative reverse transcription–PCR performed following standard procedures using gene-specific forward and reverse primers (See Supplementary Table 1). All samples were normalized to an internal control gene, β-actin, and the comparative Ct method was used to assess the relative gene expression. Samples were tested in triplicates.

### Western blot analysis of cell lines and lung tumor tissues

For the preparation of cell lysates from cell cultures, DMSO-, siRNA-, triptolide-, or HA-treated cells were incubated in 1x RIPA buffer with protease inhibitor and phosphatase inhibitor (Pierce, Rockford, IL) for 10 min on ice. For the analysis of lung tumor tissues, three pieces of tumors collected from three different lung lobes from each rat were mixed and homogenized in 1x RIPA buffer containing protease inhibitor and phosphatase inhibitor. Subsequently, cell and tissue lysates were centrifuged at 14,000 g for 10 min at 4°C, and the supernatants collected, aliquoted and stored at −80°C. Western immunoblotting analysis of the different proteins was performed following standard procedures. For each protein, at least three Western blot assays were carried out. For quantitative determination of protein levels, densitometric measurements of Western blot bands were performed using digitalized scientific software program UN-SCAN-IT software (Silk Scientific, Orem, Utah).

### Statistical analysis

All data are reported as mean ± SD of triplicate determinations. Between-group comparisons were performed using one-way ANOVA and two-tailed *t*-test in Graphpad Prism 5 software (Graphpad, La Jolla, CA). *P*-values < 0.05 were considered significant.

## SUPPLEMENTARY MATERIALS FIGURES AND TABLE



## Abbreviations

HA: hyaluronan
RHAMM: Receptor for Hyaluronic Acid-Mediated Motility
NSCLC: Non-Small Cell Lung Cancer
HAS2: Hyaluronan Acid Synthase 2
HAS3: Hyaluronan Acid Synthase 3
DPPC: 1,2-dipalmitoyl-sn-glycerol-3-phosphatidylcholine
DSPE: 1,2-distearoyl-sn-glycerol-3-phosphatidylethanolamine
