# Supplementary material for: Triptolide suppresses the in vitro and in vivo growth of lung cancer cells by targeting hyaluronan-CD44/RHAMM signaling
Source: Oncotarget. 2017 Mar 3;8(16):26927–40. doi: 10.18632/oncotarget.15879 (PMC5432308; doi:10.18632/oncotarget.15879)
Supplement: Supplementary file 1 [file oncotarget-08-26927-s001.pdf]

Triptolide suppresses the *in vitro* and *in vivo* growth of lung cancer cells by targeting hyaluronan-CD44/RHAMM signaling

Supplementary Materials

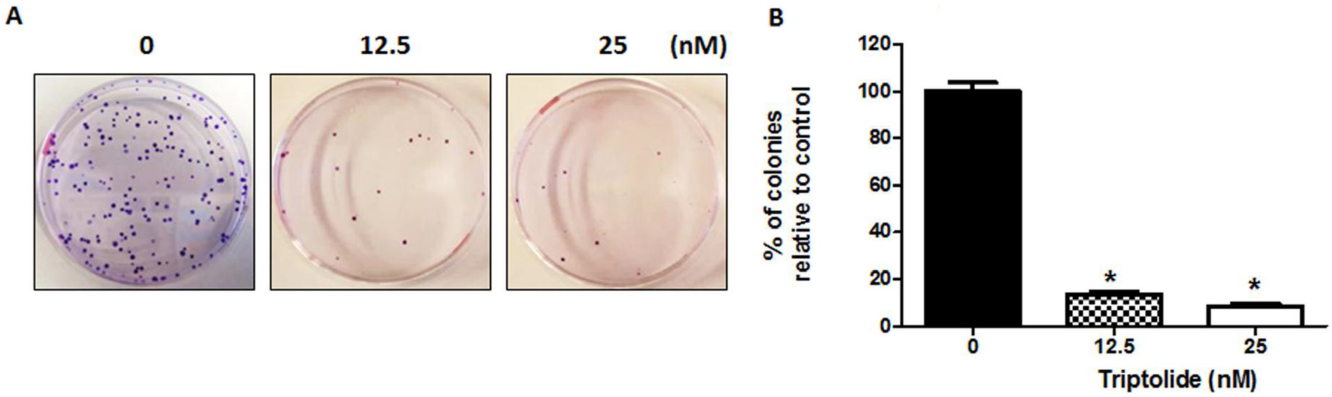

Supplementary Figure 1: Triptolide suppresses the colony formation ability of A549 cells. (**A**) Representative figures showing the dose dependent effect of triptoide on the colony formation in A549 cells. (**B**) Bar graph showing the percent of colonies in untreated- and triptolide-treated A549 cells. Cells treated with DMSO or triptolide (12.5 and 25 nM) for 24 h were seeded in 60 mm culture plates and incubated for two weeks in RPMI 1640 medium supplemented with 10% FBS in the absence of triptolide. The colonies were stained for 10 min at room temperature with 0.5% crystal violet prepared in 30% ethanol. After washing out the dye with tap water, colonies were counted. *$P < 0.05$, compared with the untreated A549 cells. Assays were performed in three times on different days.

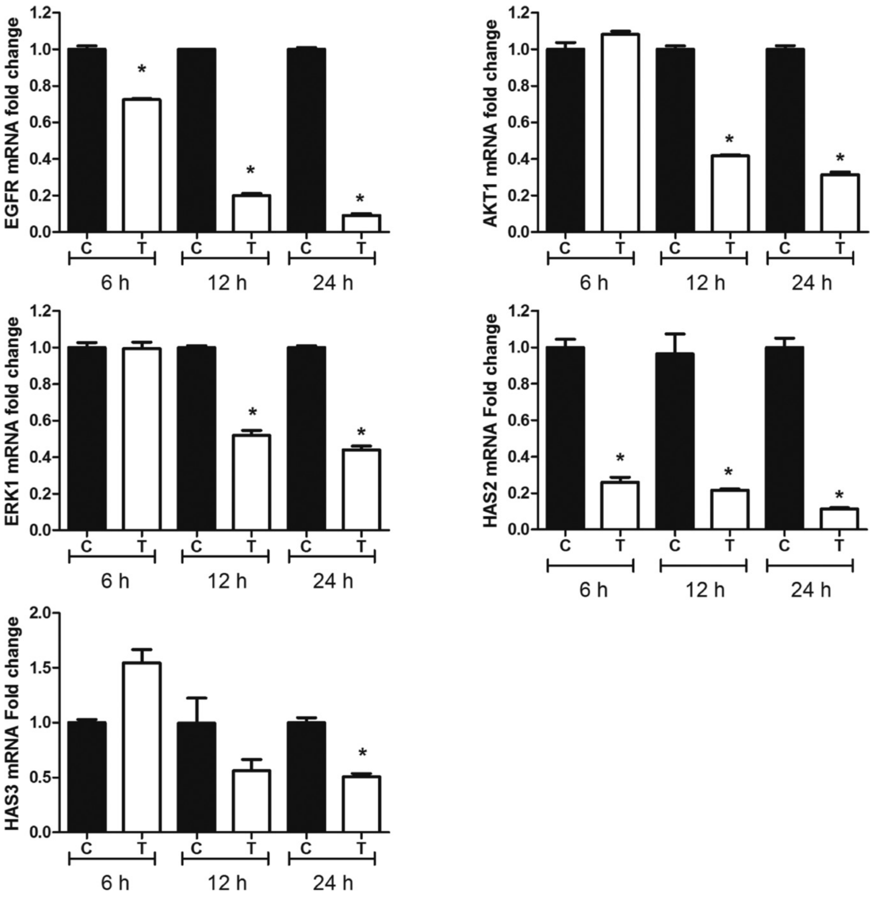

Supplementary Figure 2: Modulation of levels of HAS2, HAS3, EGFR, AKT1 and ERK in A549 cells treated with triptolide (25 nM) or DMSO for different time periods (6, 12 and 24 h). *$P < 0.05$, compared with the untreated A549 cells. C, Control (DMSO); T, triptolide

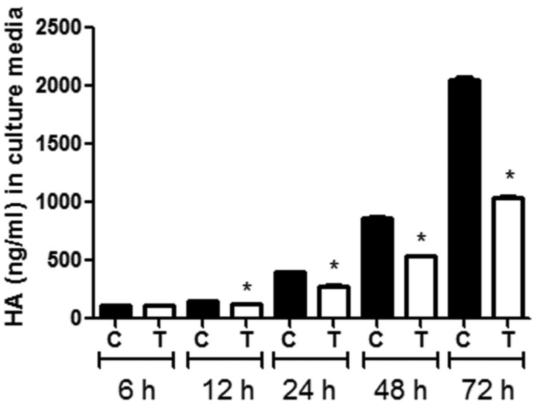

Supplementary Figure 3: HA levels in the culture media collected from A549 cells treated with triptolide (25 nM) or DMSO (control) for different time periods (6, 12, 24, 48 and 72 h). *$P < 0.05$, compared with the untreated A549 cells. C, Control; T, triptolide (25 nM).

Supplementary Table 1: Primer information used for qRT-PCR

Gene name	Forward primer (5′-3′)	Reverse primer (5′-3′)
CD44	ATCATCTTGGCATCCCTCTTG	TGAGTCCACTTGGCTTTCTG
RHAMM	AGCAACAGGAGGAAGACTTTAG	GAGGAGACGCCACTTGTTAAT
HAS1	TGTGACTCGGACACAAGGTTG	GCCTCAAGAAACTGCTGCAA
HAS2	GGGACGAAGTGTGGATTATGT	GAGATCCAGGAATCGTACTTGTT
HAS3	GCACCTTCTCGTGCATCAT	TCCAGGACTCGAAGCATCT
EGFR	GCTGGATGATAGACGCAGATAG	GAAGTTGGAGTCAGGACTTG
AKT1	CTTCTATGGCGCTGAGATTGT	GCCCGAAGTCTGTGATCTTAAT
ERK1	GGTACAGGGCTCCAGAAATTAT	TGGAAAGATGGGCCTGTTAG
β-actin	ACGGTCAGGTCATCACTATC	ACTGTGTTGGCATAGAGGTC
